# Variability in the precursor proteins of collagen I and III in different stages of COPD

**DOI:** 10.1186/1465-9921-11-165

**Published:** 2010-11-30

**Authors:** Terttu Harju, Vuokko L Kinnula, Paavo Pääkkö, Kaisa Salmenkivi, Juha Risteli, Riitta Kaarteenaho

**Affiliations:** 1 Institute of Clinical Medicine, Department of Internal Medicine, Respiratory Unit, Centre of Excellence in Research, P. O. Box 5000, 90014 University of Oulu, Oulu, Finland; 2 Department of Internal Medicine, Clinical Research Center, Oulu University Hospital, Oulu, Finland; 3 Department of Medicine, Division of Pulmonary Medicine, University of Helsinki and Helsinki University Central Hospital, Helsinki, Finland; 4 Department of Pathology, Oulu University Hospital, Oulu, Finland; 5 Department of Pathology, HUSLAB, Helsinki University Central Hospital and University of Helsinki, Helsinki, Finland; 6 Department of Clinical Chemistry, Oulu University Hospital, Oulu, Finland

## Abstract

**Background:**

Levels of precursor proteins of collagen I and III are increased in fibrotic pulmonary diseases. This study determined whether the expression of precursors of type I and III collagen proteins would be increased in small and large airways of COPD patients in various stages of the disease reflecting fibrogenesis.

**Methods:**

The levels of precursor proteins of collagen I and III were studied by immunohistochemistry and quantified by image analysis in lung tissue of 16 non-smokers, 20 smokers with normal lung function, 20 smokers with stage I-II COPD and 8 ex-smokers with stage IV COPD.

**Results:**

In large airways, the subepithelial layer which was positive for precursor proteins of collagen I and III was thicker in smokers and in stage I-II COPD compared to non-smokers. Large airways in stage IV COPD showed reduced expression of precursor protein of collagen I whereas precursor of collagen III was increased. The amount of precursor protein of collagen III was increased in small airways of smokers and stage I-II COPD but reduced in stage IV COPD.

**Conclusions:**

Precursor proteins of collagen I and III revealed different expression profiles in large and small airways in various stages of COPD. Smoking enhanced expression of both precursors in large airways with a positive correlation with pack-years.

## Background

Repeated exposure to cigarette smoke induces persistent inflammation and oxidative stress in lungs, which leads to damage of lung parenchyma and airways and a process of continuous repair and remodeling [1]. It is not clear why changes in the peripheral lung of some patients are predominantly emphysematous with loss of alveolar attachments, increased alveolar septal wall thickness [2] and decreased lung elastic recoil, while in others, thickening of the walls of small airways is the predominant feature [3,4].

The small airways are the major source of airflow resistance [5] and thus both emphysematous changes and small airway obstruction increase small airway resistance. The progression of chronic obstructive pulmonary disease (COPD) is clearly associated with a thickening of the airway wall and each of its compartments through a repair or remodeling process [3]. High resolution computed tomography (HRCT) analysis of COPD lungs has revealed that the values of forced expiratory volume in 1 second (FEV1, %predicted) correlate well with airway luminal area and, to a lesser extent, with the degree of wall thickening [6]. Degradation of airway wall elastin leads to a significant reduction of the elastin content in small airways and alveoli, and this change correlates with airflow limitation [7]. Degraded elastin is later replaced by other components of the extracellular matrix. Little is known about the composition of this newly formed extracellular matrix in the small airway wall. It has been reported that cigarette smoke activates airway epithelial cells to release mediators that trigger fibroblast activity [8]. In mice, long term exposure to cigarette smoke induced a profibrotic response in the airways but the parenchyma failed to repair damage to the matrix [9].

There are clear histopathologic differences between asthma and COPD [10,11], i.e. asthma is characterized by epithelial shedding, thickened basement membrane whereas in COPD one encounters alveolar disruption, small airway wall thickening and inflammation. However little is known about the turnover of the extracellular matrix in COPD.

Collagens are the classical components of the extracellular matrix. In lung tissue, collagen I is the most common collagen and it confers tensile properties while collagen III permits multidirectional flexibility, contributing to lung compliance [12]. Collagens are synthetized primarily by fibroblasts as precursor molecules with the propeptides being cleaved during the process of secretion of the newly formed collagens. Type I procollagen propeptides have been detected in the lungs of patients with active fibrosis, and an increased amount of mRNA coding for type I collagen in the foci of highly activated fibroblasts [13]. The amino-terminal propeptide of type III procollagen (PIIINP) has been claimed to be a marker of the synthesis of type III collagen since its concentration was elevated during wound healing [14]. The levels of PINP and PIIINP have been reported to be increased in sarcoidosis [15] as well as in the bronchoalveolar lavage fluid obtained from patients with interstitial lung diseases [16] and in developing lung [17].

Cigarette smoke induces changes in lung tissue that are not simply destructive since the smoke can also trigger an active repair process leading to protein production and small airway remodeling. It is not known whether the destruction of lung parenchyma leads to the compensatory formation of connective tissue in an attempt to preserve lung tissue integrity. The aim of this study was to evaluate the expression of precursor proteins of type I and III collagen in small and large airways of COPD patients in order to determine if the expression of these proteins would change during the progression of COPD.

## Methods

Lung tissue specimens from 56 patients (20 current smokers with COPD, 20 current smokers with normal lung function and 16 life-long non-smokers) undergoing resection for lung tumour were drawn for immunohistochemical studies from the archives of the Department of Pathology, Oulu University Hospital. Due to the fact that the resection of malignant tumors may theoretically have an influence on the adjacent structures, lung tissue specimens of non-malignant lung obtained during surgery for hamartomas were additionally included (four in the non-smoker group, one in the smoker group and one in the COPD-group). In all, 33% of the operations were pulmectomies, 60% lobectomies and 7% bilobectomies. No wedge resections were included. Tissue specimens from tumor-free central bronchi and peripheral lung tissue were selected. The patients were not receiving any corticosteroid therapy (neither inhaled nor systemic) and did not suffer from an asbestos-related disease. In addition, lung tissue from 8 patients (4 with alpha-1-antitrypsin deficiency and 4 with a normal alpha-1-antitrypsin levels) undergoing lung transplantation due to very severe i.e. stage IV COPD were obtained from the Department of Pathology, Helsinki University Central Hospital. These patients were receiving either inhaled or systemic corticosteroid treatment and they were all ex-smokers. The lungs were fixed in inflation. The size of the each lung tissue specimen was approximately 1-2 cm^2^. COPD was defined on the basis of preoperative lung function: FEV1/FVC less than 70% and no reversibility (bronchodilatation effect less than 12%). The clinical characteristics were obtained from the patient records (Table 1).

**Table 1:** Patient characteristics of the immunohistochemical samples

	Non-smokers n = 16	Smokers n = 20	COPD stage I-II n = 20	COPD stage IV n = 8	p-value
Age, years	65 (13)**	63 (8)	65 (7)	54 (8)	0.039
Sex M:F	8:8	15:5	16:4	5:3	0.096
Pack-years	0*	46 (20)	39 (14)	34 (18)	< 0.001
FEV1 l/s	2.96 (1.19)§	3.02 (0.71)	2.2 (0.54)	0.66 (0.24)	< 0.001
FEV1 %predicted	98 (15)	91 (9)#	68 (13)	21 (12)	< 0.001
FEV1 postbd	2.98 (1.1)	3.26 (0.81)	2.23 (0.38)	NA	
FVC	3.44 (1.3)	3.59 (0.91)	3.51 (1.1)	1.78 (1.0)	< 0.001
FVC postbd	3.46 (1.21)	3.79 (1.1)	3.66 (1.16)	NA	
FEV1/FVC %	86 (9)*	84 (11)#	60 (8)	37 (13)	< 0.001
DCO %predicted	91 (15)**	77 (15)§§	75 (10)	29 (8)	< 0.001
DCO/VA %predicted	89 (11)**	82 (12)§§	77 (21)	46 (11)	< 0.001

Mean (SD)

*The mean difference between non-smokers and smokers is significant at the 0.05 level, Dunnett t-test.

§ The mean difference between non-smokers and COPD-patients is significant at the 0.05 level, Dunnett t-test.

# The mean difference between smokers and COPD-patients is significant at the 0.05 level, Dunnett t-test.

** The mean difference between non-smokers and patients with severe COPD (GOLD stage IV) is significant at the 0.05 level, Dunnett t-test

§§ The mean difference between smokers and patients with severe COPD (GOLD stage IV) is significant at the 0.05 level, Dunnett t-test

### Immunohistochemistry

Formalin-fixed paraffin-embedded lung tissue specimens were identified from computerized records. All material was re-evaluated by a pulmonary pathologist and a pulmonologist. Two tissue blocks from each patient were selected, one from the resection line with central cartilage-containing bronchus and the other from the peripheral lung. Four-μm sections were cut for immunohistochemical analyses. The sections were deparaffinized in xylene and rehydrated in a descending ethanol series. Endogenous peroxidase was blocked by incubating the sections in 3% hydrogen peroxide in absolute methanol for 15 minutes.

The primary polyclonal antibodies to the amino-terminal propeptides of human type I procollagen (PINP; 0.658 ug/ul) and type III procollagen (PIIINP; 0.15 μg/μl) were produced as described previously [18,19] and used at concentrations of 1:10000 and 1:4000, respectively. Antibodies to these amino-terminal propeptides react intracellularly with the amino-terminal domains of procollagen molecules that are associated with increased collagen synthesis, and extracellularly with pN-collagen in collagen fibres (either mature or preferentially newly synthesized) in the extracellular space. Serial sections of additional cases were taken to demonstrate the co-localization of studied collagen I and III propeptides and fibroblasts, using antibodies against alpha-smooth muscle actin (1:1000) and vimentin (1:1500).

The immunohistochemical staining was performed as previously described [15,17] using the Histostain-Plus Kit (Zymed Laboratories Inc, San Francisco, CA), and the chromogen was aminoethyl carbazole (AEC) (Zymed Laboratories Inc.). In the negative controls, the primary antibodies were substituted with phosphate-buffered saline (PBS) or rabbit primary antibody isotype control from Zymed Laboratories Inc.

### Image analysis

#### Analyses of small airways

**Figure 1:**
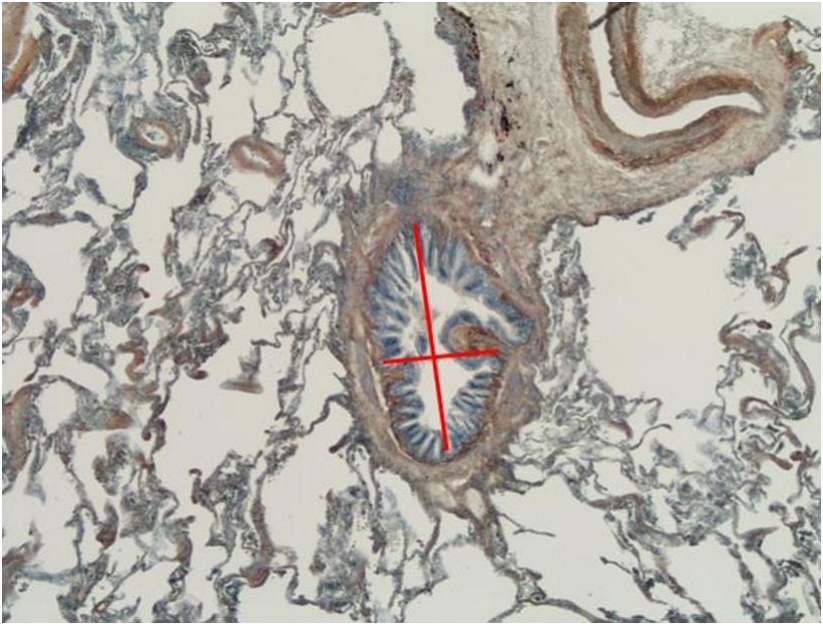
**Analysis of small airways by the image analysis method**. Short and long diameter of the bronchioles.

**Figure 2:**
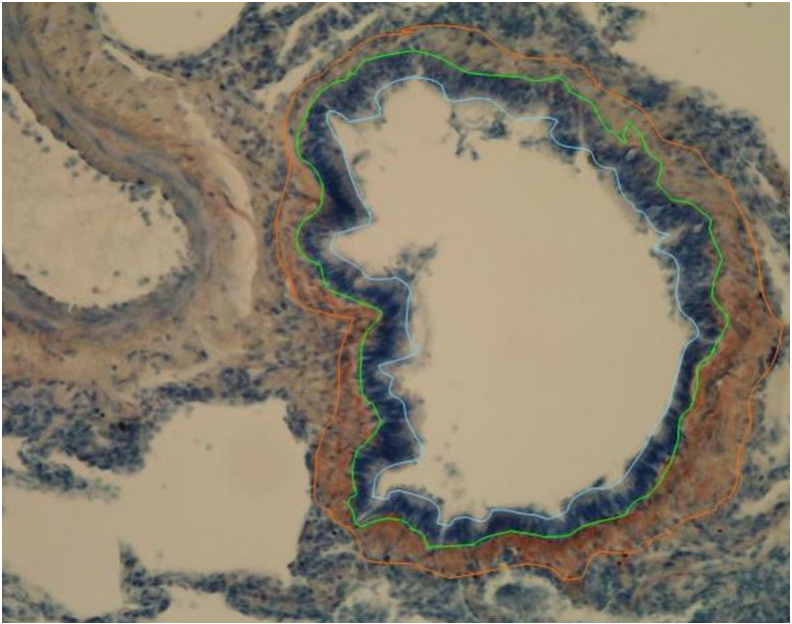
**Analysis of small airways by the image analysis method**. Measurements: internal perimeter (blue line), basement membrane perimeter (green line) and external perimeter of expression of precursor protein of collagen III (orange line).

All membranous bronchioles with diameters less than 2 mm were analyzed. Digital photographs were taken using a Leica DFC 320 camera attached to a Nikon Mikrophot SA microscope, via Leica IM 50 software. The measurements made are shown in Figures 1 and 2: these included an assessment of the diameter of the lumen, both longest and shortest dimensions; area and perimeter of the lumen bounded by respiratory epithelium; area and perimeter surrounded by the basement membrane and epithelial cell height, measured at 15 random locations. The investigator performing the morphometric airways assessments was blinded to the clinical data. The characteristics of the studied small airways are described in Table 2.

**Table 2:** Small airway characteristics

	Non-smoker n = 15	Smoker n = 32	COPD stage I-II n = 35	COPD stage IV n = 8	p-value ANOVA
Airway diameter, mm					
Longest	0.89 (0.70)	0.72 (0.36)	0.84 (0.60)	0.70 (0.40)	0.759
Shortest	0.40 (0.50)	0.26 (0.174)	0.30 (0.21)	0.07 (0.03)	0.085
Average	0.64 (0.54)	0.49 (0.24)	0.48 (0.27)	0.40 (0.21)	0.503
Internal lumen					
perimeter, mm	3.17 (2.14)	2.60 (1.45)	2.48 (1.25)	2.17 (1.04)	0.587
Area, mm^2^	0.45 (0.85)	0.17 (0.16)	0.26 (0.28)	0.09 (0.14)	0.248
Basement membrane					
Perimeter, mm	3.30 (2.25)	2.72 (1.46)	2.47 (1.22)	2.34 (1.11)	0.591
Area, mm^2^	0.56 (0.93)	0.26 (0.21)	0.29 (0.30)	0.17 (0.17)	0.239
Epithelial area, mm^2^	0.11 (0.10)	0.09 (0.08)	0.09 (0.09)	0.08 (0.06)	0.938
Thickness of airway wall within RBM** (μm)	23.3 (12.3)	26.1 (9.6)	30.9 (11.1)	22.0 (4.7)	0.029

mean (SD)

n = number of bronchioli studied

*statistically significant difference compared to stage IV COPD

**RBM = reticular basement membrane, including the membrane itself

#### Precursor proteins of collagen I and III

The immunohistochemical expression of precursor proteins of collagen I and III was quantified by the image analysis method. Immunohistochemically stained slides of central bronchi and peripheral bronchioli were photographed digitally so as to include all of the chosen material described in the previous paragraph. The extent of positive expression was measured in large and small airways in each study case. The quantification was based on a measurement of the thickness of the subepithelial band expressing precursor proteins of collagen I and III at 30 random locations and the subepithelial area expressing these precursor proteins between the basement membrane and the inner smooth muscle border. Staining was assessed with an image analysis system using freely available software ImageJ version 1.24o developed at the National Institutes of Health, using the technique described by Kim et al [20].

### Statistical methods

The statistical analyses were performed with SPSS for Windows software (SPSS, Chicago, IL, USA). Continuous data were compared using analysis of variance (ANOVA). When ANOVA results indicated that the groups differed, post hoc comparisons were performed using two-tailed t-tests. Categorical data were compared using Fisher's exact test designed for small sample groups. P-values less than 0.05 were considered statistically significant.

### Ethical considerations

The study protocol was approved by the ethical committees of the University of Oulu and Oulu University Hospital, Helsinki University Central Hospital and by the Finnish Natiolegal Board.

## Results

### Image analysis of small airways

Peripheral lung samples showed 1-3 transversely cut bronchioles (diameter < 2 mm) per case. Although bronchioles of stage IV (very severe) COPD were collapsed with a small internal lumen area, the other dimensions did not reveal any statistically significant differences between the groups (Table 2). The thickness of the airway wall within the reticular basement membrane (RBM included) was 23.3 (12.3) μm in the non-smoker group, 26.1 (9.6) μm in the smoker group, 30.9 (11.1) μm in the stage I-II (mild to moderate) COPD group and 22.0 (4.7) μm in the stage IV COPD group, the difference being statistically significant between the two COPD groups. There was a negative correlation between the diffusion coefficient and the thickness of measured compartment (r = -0.560, p = 0.010) in the COPD group but no correlations with other lung function measurements.

Immunohistochemical expression and image analysis of type I and III collagen protein precursors

**Figure 3:**
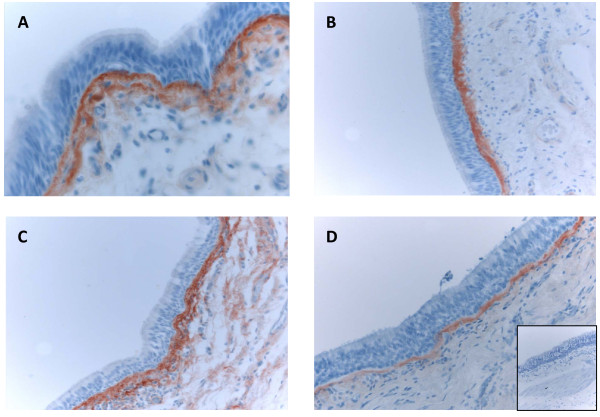
**Immunohistochemical staining for precursor protein of collagen III in bronchus of a non-smoker (A), a smoker (B), a patient with mild (stage I) COPD (C) and a patient with very severe (stage IV) COPD (D) showing that in large airways, the subepithelial layer that was positive for precursor protein of collagen III was thicker in smokers and in stage I-II COPD compared to non-smokers and patients with stage IV COPD**.

**Figure 4:**
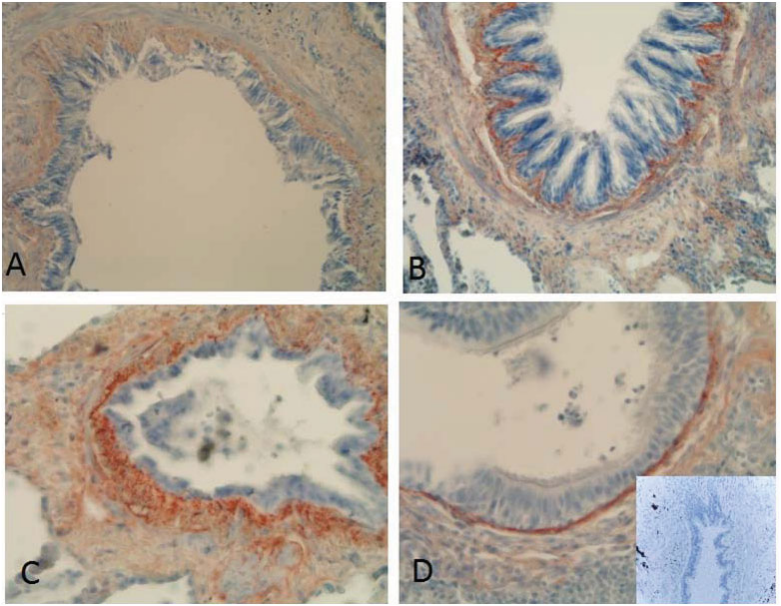
**Immunohistochemical staining for precursor protein of collagen III in small airways of a non-smoker (A), a smoker (B), a patient with stage I COPD (C) and a patient with stage IV COPD (D)**. showing increased expression of precursor protein of collagen III in small airways of smokers and stage I-II COPD, and decreased expression in stage IV COPD.

Immunohistochemical staining of the precursor protein of collagen I and III was present mainly extracellularly, being visible as linear and reticular fibers or as more intensive bands in the subepithelial layer of central bronchi as well as in the peripheral bronchioles. (Figures 3 and 4). The adventitia of vascular walls and the area demarcating chondrocytes displayed pronounced immunoreactivity. The airway epithelium itself was always negative. The alveolar epithelium was usually negative for both precursor proteins, with the exception of some cases which exhibited intense local positivity within emphysematic alveolar walls. The overall immunohistochemical expression of precursor protein of collagen III was more pronounced than that of collagen I.

#### Precursor protein of collagen I

**Figure 5:**
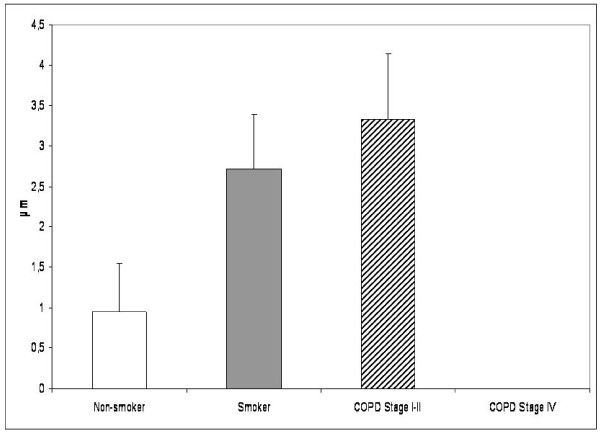
**Precursor protein of collagen I in the large airways of non-smokers, smokers and the patients with COPD**. Thickness of subepithelial layer expressing precursor protein of collagen I displayed an increase of this protein in smokers and in patients with stage I-II COPD compared to non-smokers. Its thickness was highly diminished in the patients with very severe COPD. The difference between stage I-II and stage IV COPD was statistically significant.

Only 15% of non-smoking cases exhibited immunoreactivity for the precursor of collagen I in the subepithelial layer of the central bronchus, compared to 45% of smokers and 52% of COPD-patients (p = 0.031, Pearson's Chi-Square). The precursor protein of collagen I positive subepithelial layer was thicker in the smokers and patients with stage I-II COPD when compared to the non-smokers. The patients with stage IV COPD showed diminished expression of collagen I precursor when compared with the patients with milder disease or the smokers (Figure 5). The difference between stage I-II and stage IV COPD was statistically significant. Smokers expressing positivity for precursor of collagen I in the large airways had less airway obstruction than the cases with negative expression (FEV1% predicted 79% vs 66%, p = 0.045; MEF50% predicted 65% vs 43%, p = 0.014). The subepithelial layer of bronchi expressing precursor protein I was thicker in smokers compared to the corresponding situation in non-smokers (2.74 μm vs 0.95 μm, p = 0.049). In smokers, the correlation between the positive layer thickness and pack years was weakly positive (r = 0.255, p = 0.039). No correlation was found between the lung functions and the expression of precursor protein of collagen I in the subepithelial layer. The subepithelial layer of small bronchioles did not exhibit any positivity for the precursor protein of collagen I.

#### Precursor protein of collagen III

**Figure 6:**
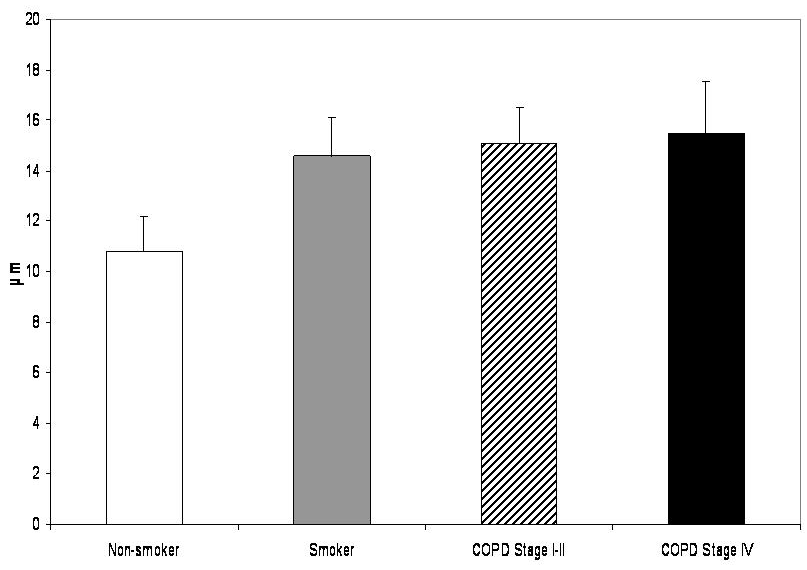
**Precursor protein of collagen III in the large airways of non-smokers, smokers and the patients with COPD**. The subepithelial layer expressing precursor protein of collagen III was thicker in all smoker groups compared to non-smokers.

**Figure 7:**
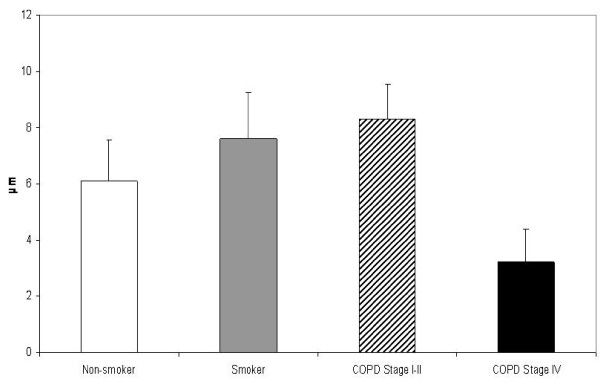
**Precursor protein of collagen III in the small airways of non-smokers, smokers and the patients with COPD**. The subepithelial layer expressing precursor protein of collagen III was thicker in smokers and in the patients with stage I-II COPD compared to the non-smokers but its accumulation was decreased in patients with stage IV COPD. The difference between stage I-II and stage IV COPD was statistically significant (p = 0.015).

**Figure 8:**
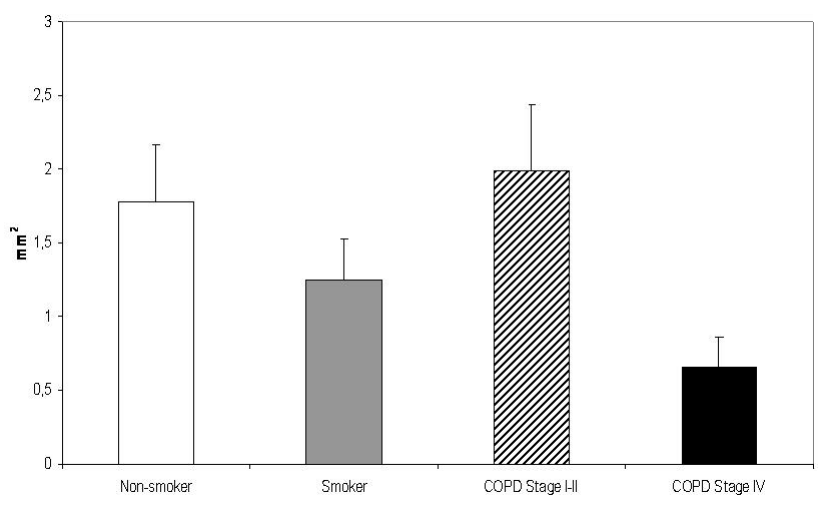
**Precursor protein of collagen III in the small airways of non-smokers, smokers and the patients with COPD**. The subepithelial area with positive staining for precursor protein of collagen III was thinner in severe COPD compared to non-smokers (p = 0.017), smokers (p = 0.045) and patients with stage I-II COPD (p = 0.012).

The subepithelial layer of large airways expressing precursor protein of collagen III was thicker in smokers than in non-smokers, the exact measures being 14.97 μm vs 10.82 μm,(p = 0.019). (Figure 6) The subepithelial layer thickness displayed a positive correlation with pack-years in healthy smokers (r = 0.519, p = 0.016) as well as in COPD-smokers (r = 0.346, p = 0.007). In addition, the patients with stage IV COPD displayed increased expression of precursor protein of collagen III in their large airways. In bronchioles i.e. in small airways, the staining of precursor protein of collagen III was increased in the smokers and in these cases the thickness of the subepithelial layer expressing precursor of collagen III protein correlated positively with pack-years (r = 0.319, p = 0.024). In the patients with COPD, the amount of precursor protein of collagen III correlated with pack-years (r = 0.497, p = 0.022), FEV% predicted (r = 0.549, p = 0.010) and DCO/VA (r = 0.471, p = 0.042). The COPD-patients in stage I-II displayed increased expression of collagen III precursor protein in their small airways, but this was declined in the patients with stage IV disease. (Figure 7) The exact values of the precursor protein of collagen III expressing subepithelial layer thicknesses in bronchioles were 6.51 μm in non-smokers, 6.87 μm in smoker group, 8.49 μm in the patients with stage I-II COPD and 2.87 μm in the patients with stage IV COPD. The difference between stage I-II and stage IV COPD groups was significant at the 0.05 level. The subepithelial area with positive collagen III precursor staining was smaller in patients with stage IV COPD as compared to non-smokers (p = 0.017), smokers (p = 0.045) and those patients with stage I-II COPD (p = 0.012) (Figure 8).

### The co-localization study

**Figure 9:**
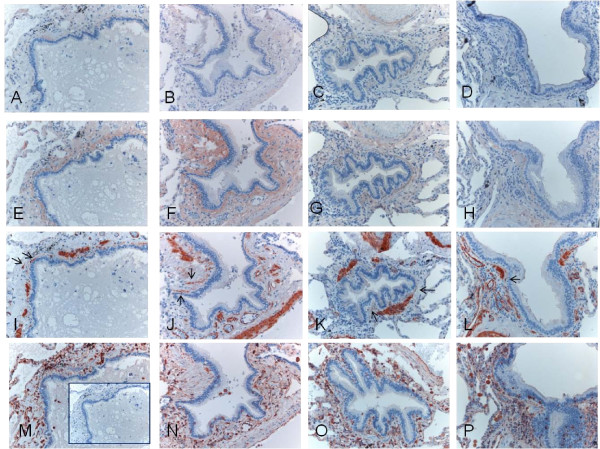
**Spindle-shaped, alpha-smooth muscle actin positive fibroblastic cells were found to co-localise with collagen III propeptides in serial sections of peripheral bronchioli**. Almost no expression of procollagen I was seen (A-D), with variable expression of collagen III propeptide (E-H) and co-localization of spindle-shaped alpha-smooth muscle actin positive fibroblastic cells (I-L, arrows), with immunohistochemistry for vimentin (M-P, M with negative PBS-control).

**Figure 10:**
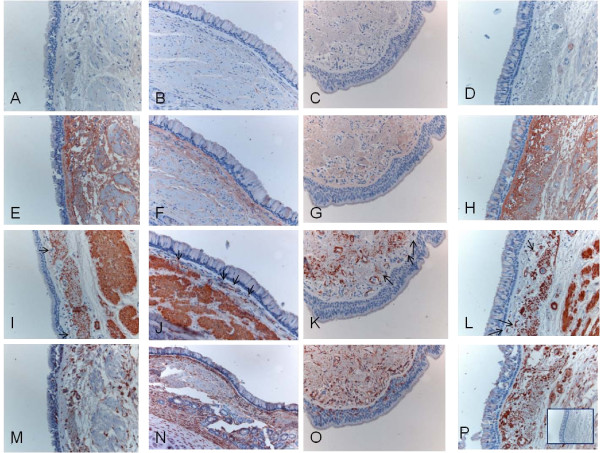
**Spindle-shaped, alpha-smooth muscle actin positive fibroblastic cells were found to co-localise with collagen III propeptides in serial sections of central bronchi**. Almost no expression of collagen I propeptides was seen (A-D), but there was variable expression of collagen III propeptide (E-H) and co-localization of spindle-shaped alpha-smooth muscle actin positive fibroblastic cells (I-L, arrows), with immunohistochemistry for vimentin (M-P, P with negative anti-rabbit-control).

The expression of propeptides for collagen I and III was found to co-localise with spindle-shaped, alpha-smooth muscle actin positive, vimentin negative fibroblastic cells in small airways (Figure 9) and large airways (Figure 10).

## Discussion

This is the first study to investigate the immunohistochemical expression of precursor proteins of collagens I and III as quantified by an image analysis technique in non-smokers, smokers and patients with COPD at various stages of the disease. The levels of both proteins were increased in large airways of smokers and patients with stage I-II COPD. In contrast, precursor proteins were expressed differently in large airways of patients with stage IV COPD i.e. in these patients the amount of precursor protein of collagen I was reduced whereas that of collagen III was elevated. The present study also revealed that the increase of the precursor protein of collagen I and III in the large airways of smokers correlated positively with pack-years. No detectable expression of the precursor protein of collagen I was found in small airways, whereas the amount of the precursor protein of collagen III was increased in the bronchioles of smokers and in stage I-II COPD. Overall, the amounts of precursors of collagen I and III were increased in smokers and stage I-II COPD but tended to decline either in large or small airways in stage IV COPD.

The accumulation of the precursor protein of collagen I could represent the synthesis of type I collagen which in turn offers protection from the distension and shearing effects of hyperinflation, while its absence may further contribute to alveolar wall disruption and emphysema formation in conjunction with other mechanisms. In contrast, the expression of the precursor protein of collagen III within small airways was increased in patients at stages I-II COPD and moreover, its amount correlated positively with the results of the lung function tests. The very thin subepithelial layer expressing collagen III precursor proteins in stage IV COPD may be attributable to decreased fibrotic activity in small airways in end-state COPD, and the combination of this defective repair of injured airways with elastin destruction which could evoke diminished compliance and disrupt the elastic properties of lung tissue. Decreased precursor protein of collagen III in stage IV COPD may also be due to the end-stage of the remodelling process, a diminished capacity of the lung cells to produce collagen III, and/or a shift in the balance towards degradation. The discrepancy in the expression profiles of the precursor proteins of collagens I and III reflects the fact that the regulative processes of these two collagens differ in COPD. In addition, there were few spindle shaped alpha-smooth muscle actin positive fibroblastic cells especially in the small airways but these cells co-localised with the expression of the studied collagen propeptides, suggesting that fibroblasts could well be one source of this newly synthesised collagen.

We have previously shown that in the developing human lung, the precursor proteins of collagen I and III were expressed through all gestational ages and also in respiratory distress syndrome and bronchopulmonary dysplasia in a rather similar way both in bronchi and in the bronchioles underneath the airway epithelium [17] Thus, finding a difference between the expression of precursor I and III in the small airways of patients with COPD was somewhat surprising. This may indicate that there is a different kind of fibrotic process in COPD compared to that occurring in other fibrotic lung diseases. Furthermore, we have investigated the expression of the precursors of collagens I and III in idiopathic pulmonary fibrosis (IPF)/usual interstitial pneumonia (UIP); in that disease, precursor protein of type I was mostly present as intracellular spots in the newly formed fibrosis while the precursor protein of type III was expressed underneath the metaplastic alveolar epithelium [15]. In general, mRNAs of both collagens have colocalized with the precursor proteins [13,17]. The most abundant cell type displaying positivity for mRNAs seemed to be the myofibroblast. One could speculate that different subpopulations or phenotypes of fibroblasts are involved in the fibrogenesis in different lung disorders.

The collagen III deposition which was accompanied by an excess of fibroblasts as detected in the large airways of patients with severe asthma was not observed in COPD [21]. The myofibroblasts which are often seen in asthma with subepithelial fibrosis [22], have not been studied in COPD. The exact producer of this remodeled matrix as well as the role of inflammatory cells and activated epithelium in the remodeling process and in fibroblast activation/differentiation into myofibroblasts in small airway fibrosis in COPD is unclear. Fibroblasts from COPD-patients are known to have a reduced capability to repair injured tissue [23]. In addition, it is not known either whether or not myofibroblasts persist in the tissue and are responsible for fibrosis via increased matrix synthesis and contraction of the tissue, as is the case in fibrotic lung disorders [24]. Equally it is unclear if the peribronchial excess in connective tissue represents a disordered non-functional regional response to inflammation and oxidative stress or whether the small airway fibrosis is a compensatory phenomenon to the increased collapsibility secondary to the loss of alveolar attachments.

In our earlier studies, we found diminished levels of the rate-limiting enzyme of glutathione synthesis in smokers' lung [25] and this probably contributes to the progression of lung injury. The levels of some antioxidant enzymes are known to be increased in the airways of smokers [26], reflecting the complexity of the oxidant-antioxidant balance in the pathogenesis of COPD. The oxidant-antioxidant imbalance in turn has multiple effects on the levels and activation of growth factors. Under *in vitro *conditions cigarette smoke can inhibit fibroblast recruitment, proliferation and extracellular matrix contraction [27] as well as evoking reversible DNA damage [28]. The density of fibroblasts has been shown to be critical for TGFβ activation by cigarette smoke [29]. It has been reported that cigarette smoke releases active TGFβ1 from tracheal explants without the need for exogenous inflammatory cells and causes upregulation of connective tissue growth factor and upregulation of procollagen gene expression [30].

The limitations to this study include the lack of stage III COPD patients, since they have advanced airflow obstruction and operative treatment for their lung tumors is often not possible. Patients in the stage IV COPD groups were younger than those in the other groups, they were ex-smokers and they were heavily medicated often with both inhaled and systemic corticosteroids. Half of the patients with stage IV COPD had alpha-1-antitrypsin deficiency while the other half had normal levels of alpha-1-antitrypsin; however the histopathological destruction of lung tissue showed no difference between these two groups. In addition, no age-related change in the collagen content of the lungs of non-smokers has been found [31]. Little is known about the antifibrotic effect of steroids in COPD or the effect of smoking cessation on fibrogenesis in COPD. Cigarette smoke inhibits TGFβ release from airway epithelial cells [32] and in COPD an aberrant responsiveness of fibroblasts to cigarette smoke extract has been reported [33] but the net effect on fibrogenesis is unclear. Since the nature of this study is retrospective, and proper quantification of histological changes would require a multilevel sampling design, the problem of selection bias cannot be excluded. Thus, studies with measurement of a reference volume of entire lung and a random sampling design will be needed to confirm our results. Further studies will be needed to investigate these proteins in various subtypes of COPD i.e. airway or emphysema predominant disease. The number of peripheral bronchioli was low in stage IV COPD. This finding is new and could even be related to the progression of COPD.

Procollagen I and III are not specific to any fibrotic disease, since these proteins are components of both normal and enhanced remodeling. Our study confirms earlier reports in COPD patients that there is actual fibrosis beneath the basement membrane with increased collagen accumulation [34]. The differences between large and small airways confirm previous findings that remodeling changes are present also in the large airways [35], and moreover, this phenomenon is present in the large airways before the obstruction has developed. The pathogenetic changes noted in the small airways of COPD may represent local wound healing of injured epithelium rather than a disease of uncontrolled fibrosis of the airways.

We conclude that smoking induces immunohistochemical expression of precursor proteins of collagen I and III in large airways of healthy and diseased lung and this change correlates with the pack-years. Moreover the amounts of both precursors exhibit variable expression profiles in large and small airways of patients with COPD in various stages of the disease. Accumulation of precursor protein of collagen III is increased in the small airways of patients with mild-moderate COPD but declines in end-state disease, possibly as a marker of the cessation of active fibrogenesis or as a result of enhanced degradation of the protein. These results suggest that smoking can induce the fibrogenesis of airways even in 'healthy' smokers with normal lung function. Furthermore, in COPD not only the small airways, but also the large airways display evidence of fibrogenesis. The effective treatment of the small airway disease in COPD will require a better understanding of the relationship between airway fibrosis and airflow obstruction, and also an awareness of extracellular matrix turnover and its regulation in smoking related diseases.

## Competing interests

The authors declare that they have no competing interests.

## Authors' contributions

TH participated in the design of the study and selection of patient material, performed the morphometric analysis, the statistical analysis and drafted the manuscript. VLK participated in the design and coordination of this study, selection of patient material, and helped to draft the manuscript. PP participated in the selection of patient material and analysis of immunohistochemical results, and helped to draft the manuscript. KS participated in the selection of patient material and helped to draft the manuscript. JR participated in the design of the study and provided the collagen precursor antibodies and helped to draft the manuscript, RK conceived the study, participated in the design of the study and selection of patient material, analysis of immunohistochemical results and helped to draft the manuscript. All authors have read and approved the final manuscript.

## Funding

This work was supported by grants from the Finnish Anti-Tuberculosis Association Foundation, Finnish Association of Respiratory Medicine, Sigrid Juselius Foundation, the Academy of Finland, EVO Funding of the Helsinki University Central Hospital and Oulu University Hospital, and the Jalmari and Rauha Ahokas Foundation.
